# Angiogenic stem cell delivery platform to augment post-infarction neovasculature and reverse ventricular remodeling

**DOI:** 10.1038/s41598-022-21510-y

**Published:** 2022-10-20

**Authors:** Hye Sook Shin, Akshara Thakore, Yuko Tada, Albert J. Pedroza, Gentaro Ikeda, Ian Y. Chen, Doreen Chan, Kevin J. Jaatinen, Shin Yajima, Eric M. Pfrender, Masashi Kawamura, Phillip C. Yang, Joseph C. Wu, Eric A. Appel, Michael P. Fischbein, YJoseph Woo, Yasuhiro Shudo

**Affiliations:** 1 Department of Cardiothoracic Surgery, Falk Cardiovascular Research Center, Stanford University School of Medicine, Stanford, CA 94305 USA; 2 Department of Medicine, Division of Cardiovascular Medicine, Stanford University School of Medicine, Stanford, USA; 3 Department of Chemistry, Department of Materials Science & Engineering, Stanford University, Stanford University, Stanford, USA; 4 Stanford Cardiovascular Institute, Stanford University School of Medicine, Stanford, USA; 5 Department of Materials Science & Engineering, Department of Bioengineering, Department of Pediatric (Endocrinology), Stanford University, Stanford, USA

**Keywords:** Medical research, Translational research

## Abstract

Many cell-based therapies are challenged by the poor localization of introduced cells and the use of biomaterial scaffolds with questionable biocompatibility or bio-functionality. Endothelial progenitor cells (EPCs), a popular cell type used in cell-based therapies due to their robust angiogenic potential, are limited in their therapeutic capacity to develop into mature vasculature. Here, we demonstrate a joint delivery of human-derived endothelial progenitor cells (EPC) and smooth muscle cells (SMC) as a scaffold-free, bi-level cell sheet platform to improve ventricular remodeling and function in an athymic rat model of myocardial infarction. The transplanted bi-level cell sheet on the ischemic heart provides a biomimetic microenvironment and improved cell–cell communication, enhancing cell engraftment and angiogenesis, thereby improving ventricular remodeling. Notably, the increased density of vessel-like structures and upregulation of biological adhesion and vasculature developmental genes, such as *Cxcl12* and *Notch3*, particularly in the ischemic border zone myocardium, were observed following cell sheet transplantation. We provide compelling evidence that this SMC-EPC bi-level cell sheet construct can be a promising therapy to repair ischemic cardiomyopathy.

## Introduction

Cardiovascular diseases (CVD) are one of the predominant causes of death globally, responsible for 19.05 million deaths in 2020^1^. In particular, ischemic heart disease is the most prevalent cause of death within the spectrum of cardiovascular diseases and accounted for 41.3% of the total CVD deaths in the United States in 2019. Unfortunately, despite remarkable recent efforts to develop interventional and pharmacological treatments to mitigate the risk of ischemic heart disease^2,3^, some patients fail to respond to these and return to invasive treatments, such as percutaneous coronary intervention and coronary artery bypass graft. However, these mainstay therapies leave many patients with incomplete revascularization and often lead to congestive heart failure by cardiomyocyte injury and dysfunction, adverse ventricular remodeling, and progressive functional deterioration^4,5^. Therefore, there is an urgent need for novel therapies that harness the microcirculation-restoring capacity of the heart.

In recent years, the growing expertise in cell isolation and handling has made stem cells a good resource for developing strategies for cardiac repair. One strategy involves directly injecting cells or biodegradable polymers containing cells, growth factors, or other cytokines. However, the cells directly injected through a needle into the myocardium readily aggregate and undergo necrosis due to the mechanical stresses experienced during ejection^6^. In addition, the injected cells or other materials are poorly localized on the myocardium of interest and face rapid retention loss in the ischemic microenvironment, limiting their targeted therapeutic effects^7^. The engineered cardiac patch scaffold made from synthetic or natural biological materials exhibits improved mechanical support but with some limitations. Despite their affordability and ease of preparation, synthetic biodegradable materials (e.g., PCL, PLA, PGS, and PLGA) require functionalized modifications to provide the desired biological stimuli or adhesion to endogenous and grafted cells^8^. On the other hand, natural materials (e.g., collagen, alginate, matrigel, and fibrin) or decellularized extracellular matrix (ECM) can induce biological stimulation that synthetic materials lack, yet they require elaborate purification processes. Overall, the use of biomaterial scaffolds is still challenged by their inability to mimic the natural properties of tissues and the cause of undesirable arrhythmogenicity, toxicity, and immune rejection toward xenogeneic materials^9,10^. To avoid these drawbacks, a strategy to create scaffold-free cell sheets has been developed^11–13^. The cell sheets can be made on a culture dish engineered with a temperature-responsive polymer, poly-N-isopropylacrylamide, that changes its chemical properties from hydrophobic to hydrophilic when the temperature is lowered. The significant advantage of the cell sheet made of densely adherent cells is that the retained ECM helps the cells stay intact as a sheet without requiring an artificial scaffold, makes them easy to handle, and most importantly, helps them adhere to the surface of the host tissue, improving targeted biological effects.

Among the many types of cells used in cell-based therapies, endothelial progenitor cells (EPCs), the precursors of blood vessels, are one of the popular choices due to their robust angiogenic potential. However, these endothelial cells must be supported by smooth muscle cells (SMCs) to form mature, functional vasculature. Each cell type uniquely contributes to vascular homeostasis, and their interaction is critical for maintaining mature vessel tone and repair and remodeling associated with neovascularization^14,15^. Our previous studies showed that a rat bone marrow-derived, bi-level cell sheet of endothelial progenitor cells (EPCs) and smooth muscle cells (SMCs) improved post-infarction ventricular remodeling. Specifically, delivering a scaffold-free, bi-level cell sheet construct that maintains interactions between EPCs and SMCs enhanced neovascularization within the border zone of ischemic myocardium and minimized post-infarction adverse remodeling, improving ventricular function in a rat model of ischemic cardiomyopathy^16,17^. Our group also previously developed a simple method to trans-differentiate bone marrow-derived mesenchymal stem cells (MSCs) to “smooth muscle cell-like cells (SMCs)” to improve study practicality and translatability. Based on these previous findings, in this study, we attempted to construct a bi-level cell sheet using human-origin EPCs and transdifferentiated SMCs from MSCs. We hypothesized that the joint delivery of EPCs and SMCs as a bi-level cell sheet would enhance cell engraftment and neovascularization in the recipient myocardium, limiting post-infarction ventricular remodeling in an athymic rat model of myocardial infarction. We aim to continue this line of work toward advancing potential translational therapeutics.

## Results

### Characterization of EPCs and transdifferentiated SMC lineage

**Figure 1:**
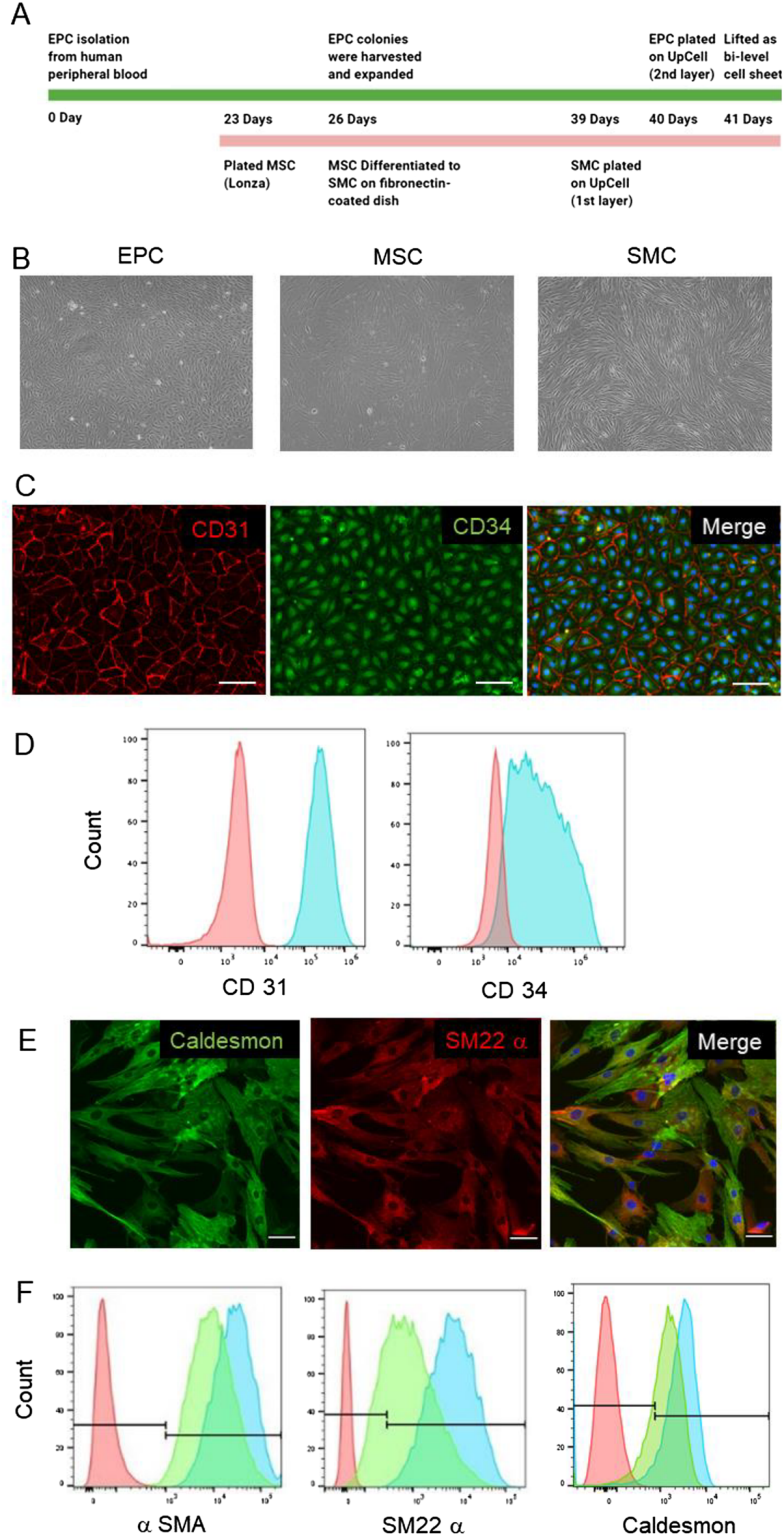
Characterization of endothelial progenitor cells (EPCs), smooth muscle cells (SMCs), and SMC-EPC bi-level cell sheets. **(A)** SMC-EPC bi-level cell sheet manufacturing protocol. **(B)** Morphologies of confluent human peripheral blood-derived EPCs (left), human bone marrow-derived MSCs (middle), and the transdifferentiated SMCs from MSCs (right). **(C)** Immunocytochemistry demonstrated CD31 (red) and CD34 (green) on EPCs. The cell nuclei were counterstained with 4′,6-diamidino-2-phenylindole (DAPI, blue). Scale bar = 50 μm. **(D)** Representative flow cytometry histogram of cultured EPCs. Red indicates unstained cells as negative control; blue, EPCs. **(E)** Immunocytochemistry demonstrated smooth muscle protein 22-alpha (SM22-α, red) and caldesmon (green) on SMCs. The cell nuclei were counterstained with DAPI (blue). Scale bar = 50 μm. **(F)** Representative flow cytometry histogram of cultured SMCs. Red indicates unstained cells as negative control; blue, transdifferentiated SMCs; green, primary human-derived SMCs as a positive control. MSC, mesenchymal stem cell; α-SMA, alpha-smooth muscle actin.

The protocol used to manufacture the SMC-EPC bi-level cell sheets is illustrated in Fig. 1A. EPCs isolated from human peripheral blood mononuclear cells exhibited typical endothelial cobblestone morphology (Fig. 1B). The isolated EPCs showed the co-localization of CD31 and CD34 (Fig. 1C), and flow cytometry immunophenotyping further confirmed these phenotypic expressions (CD31 (99.9% ± 0.03%) and CD34 (81.1% ± 8.2%)) (Fig. 1D). MSC-derived SMCs exhibited SMC-like elongated, spindle-shaped morphology compared with undifferentiated cells (Fig. 1B). Immunofluorescent staining showed the co-localization of SM22a and caldesmon on these SMCs (Fig. 1E). Flow cytometry analysis confirmed this result and showed that the transdifferentiated SMCs were highly positive for α-SMA (100.0% ± 0.0%), SM22-α (99.9% ± 0.1%), and caldesmon (89.5% ± 2.7%), showing comparable SMC marker level compared with in-vitro cultured human aortic SMCs (Fig. 1F). These results demonstrate the differentiation efficiency of EPCs and SMCs in our protocols that are consistent with previous reports^15–17^.

### The enhanced EPC vasculogenic potential using SMC-conditioned culture medium

**Figure 2:**
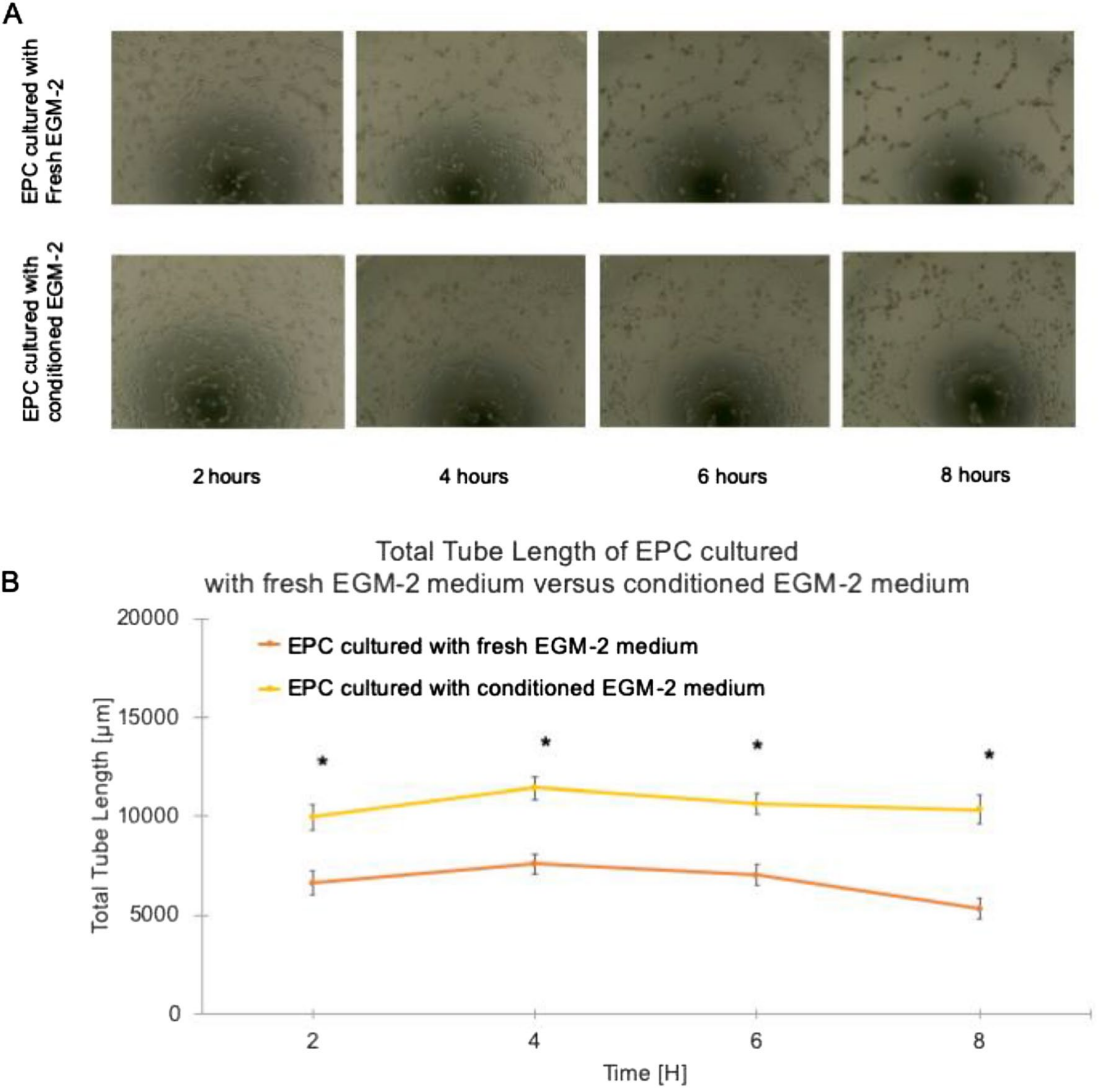
In-vitro quantification of angiogenesis by Matrigel tube formation assay. Representative images demonstrating enhanced angiogenesis of endothelial progenitor cells (EPCs) in the conditioned endothelial cell growth medium (EGM)-2 mediated by SMC compared with that in the fresh EGM-2 control. A statistically significant increase in vessel length was evident in the conditioned EGM-2 medium group compared with that in the fresh EGM-2 medium control (n = 24 in each).

The in-vitro angiogenic activity of isolated EPCs was determined by Matrigel-tube formation assay. The capacity of EPCs to induce blood-vessel-like structures was assessed by the total tube length over 8 h. During the observation period of 2–8 h, a profound increase in total tubing in both groups was observed (Fig. 2A). However, EPCs cultured in the SMC-conditioned EGM-2 medium showed enhanced angiogenic potential with a more significant number and length of vessels and branches formed than in the non-conditioned medium (Fig. 2B). This result suggests the direct SMC-mediated angiogenic potential of EPCs via paracrine signaling.

### Creation and characterization of SMC-EPC bi-level cell sheets

**Figure 3:**
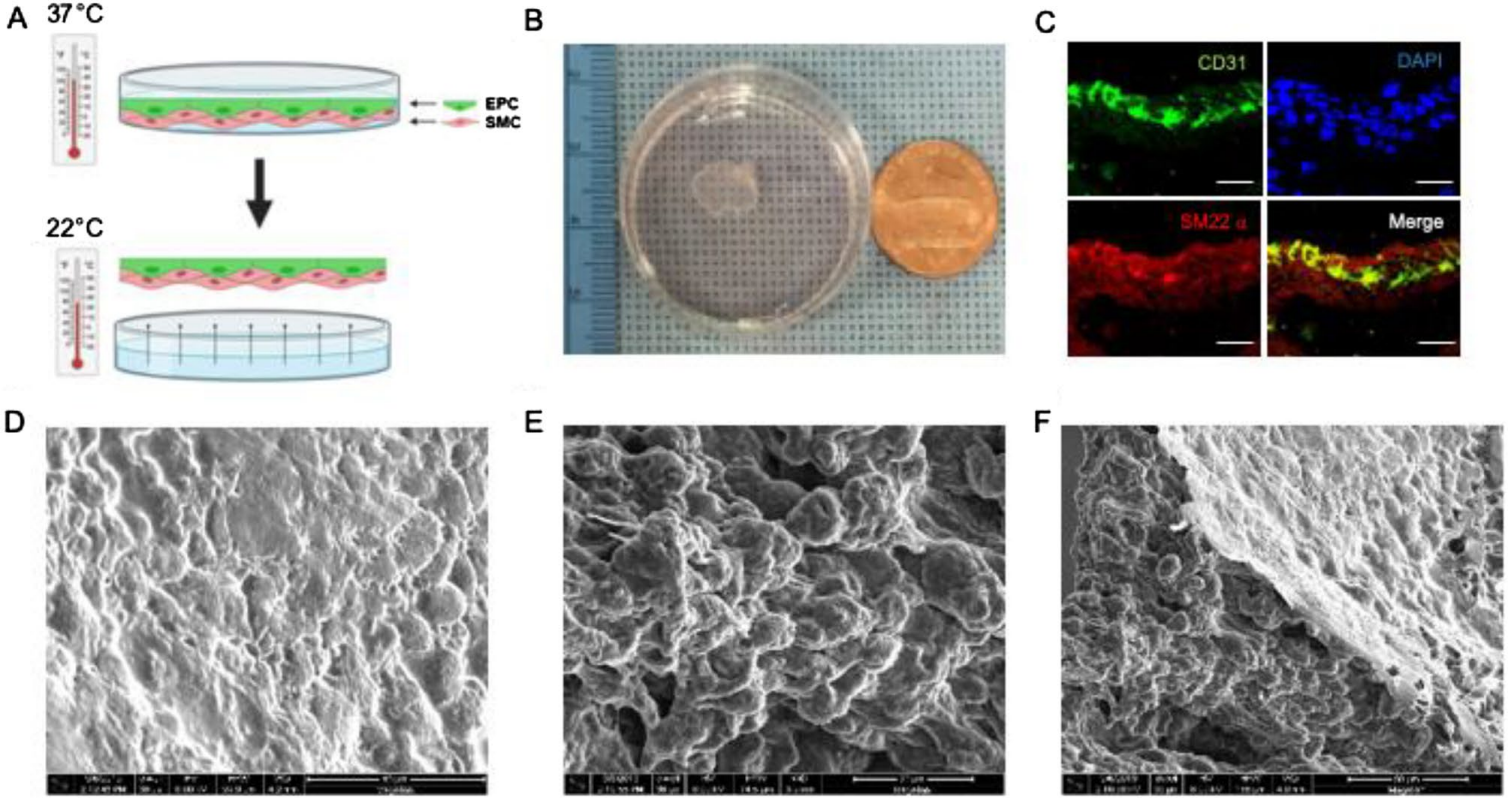
Creation and characterization of the smooth muscle cell (SMC)-endothelial progenitor cell (EPC) bi-level cell sheet. **(A)** The diagram shows the methods for engineering the cell sheet. Cell-sheet is harvested from the temperature-responsive culture dish. **(B)** A round-shaped scaffold-free SMC-EPC bi-level cell sheet in a 35-mm dish. **(C)** The bi-level cell sheet maintained CD31 positive EPC and SM22-α positive SMC in separate layers. Green indicates CD31; red, SM22-α. The cellular nuclei were counterstained with 4′,6-diamidino-2-phenylindole (DAPI, blue). Scale bar = 50 μm. **(D-F)** Representative scanning electron microscope (SEM) images of SMC-EPC bi-level cell sheet. **(D)** The examination of the surface (EPC layer) of the SMC-EPC bi-level cell sheet suggested that EPCs form a smooth confluent layer with cell borders in close contact with each other, thereby forming a thin film-like layer on top of the SMC. **(E)** The examination of the bottom (SMC layer) of the SMC-EPC bi-level cell sheet indicated that SMC morphology varies regionally from spherical to flattened, suggesting that SMCs differentiated into an elongated and spindle-shaped dense sheet-like construct with cellular extensions forming a densely adherent layer. **(F)** The interface between the two layers of the SMC-EPC bi-level cell sheet (SMC-EPC border-zone) showed the development of a cross-linked structure.

The schema used to engineer the SMC-EPC bi-level cell sheets is illustrated in Fig. 3A. Cell sheets were created on an enzymatic-free, temperature-responsive culture dish. Upon decreasing the temperature from 37 °C to 20–22 °C, the cell sheets were spontaneously detached from the culture dish (Fig. 3B). The phenotypical characteristics of the bi-level cell sheets were evaluated by immunostaining (Fig. 3C) and scanning electron microscopy (SEM) (Fig. 3D–E). SEM revealed the presence of densely adherent cells without an artificial scaffold in the cell sheet. The distinct morphologies were noted between individual cells, different cell types, and the upper and lower levels of the cell sheets. The EPCs on the upper EPC layer of the bi-level cell formed a smooth, thin confluent cell layer (Fig. 3D). On the other hand, the SMC morphology in the lower SMC layer varied regionally from spherical to flat (Fig. 3E), suggesting their morphological changes into elongated and spindle-shaped with cellular extensions to form a densely adherent layer. The interface between the two cell layers, or the SMC-EPC border zone, showed the development of a cross-linked structure (Fig. 3F).

### SMC-EPC bi-level cell sheets improved cardiac function, viability, and remodeling

**Figure 4:**
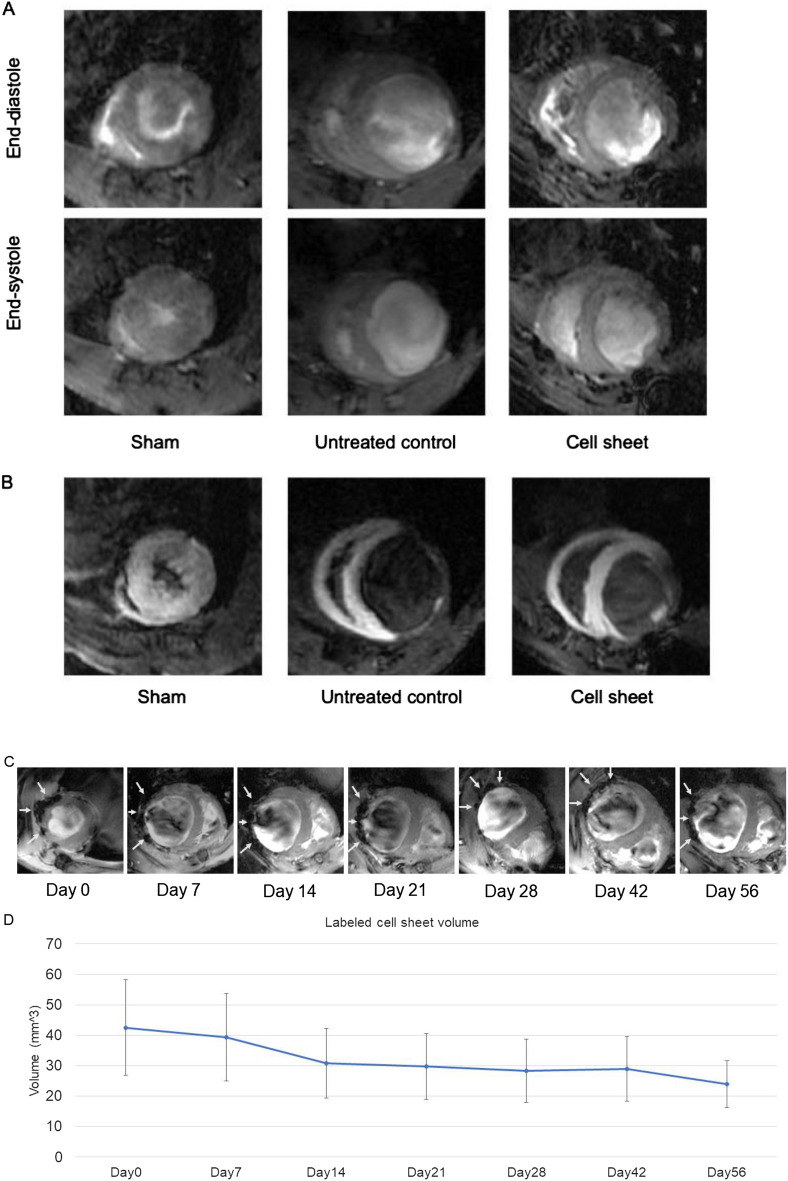
Cardiac magnetic resonance image (MRI) and manganese-enhanced magnetic resonance image (MEMRI) to evaluate cardiac function, left ventricular (LV) dimension, LV mass, and myocardial viability. **(A)** Representative cardiac MRI at end-diastolic and end-systolic phases for cell sheet-treated (n = 15), untreated control (n = 23), and sham control (n = 20) groups. Examinations were performed 8 weeks after myocardial infarction. **(B)** Representative MEMRI for cell sheet-treated (n = 15), untreated control (n = 23), and sham control (n = 20) groups. Examinations were performed 8 weeks after myocardial infarction. Ultrasmall superparamagnetic iron oxide (USPIO)-enhanced MRI to assess cell engraftment of transplanted donor cells. **(C)** Representative hypointense lesions of iron-labeled cell sheet transplantation site at 0 (baseline), 7, 14, 21, 28, 42, and 56 d after treatment. Short-axis magnetic resonance imaging showing hypointense lesions (white arrow) caused by the transplanted cell sheet adjacent to the anterior surface of the left ventricle. **(D)** Table of longitudinal change of cell engraftment ratio following cell-sheet transplantation. In the period of 7 d after cell-sheet transplantation, the cell engraftment ratio was maintained as high as 92.4% of baseline. Next, regression of cell engraftment was found, and then the ratio decreased to 72.5% of baseline at 14 d after the treatment. During the period of 14 to 42 d, this ratio was maintained at approximately 70% of the baseline. The cell engraftment ratio at 56 d was still as high as 56.3% of baseline.

The effects of bi-level cell sheet transplantation on cardiac function were assessed in an athymic rat model of ischemic cardiomyopathy using cardiac magnetic resonance imaging (MRI) and manganese-enhanced MRI (MEMRI) (Fig. 4). Representative cardiac MRI at end-systolic and end-diastolic phases and MEMRI images are shown in Fig. 4. Compared to sham animals, MI animals showed a significantly decreased left ventricular (LV) ejection fraction with concomitant increases in LV end-systolic and end-diastolic volume 8 weeks after infarction, which are all characteristics of chronic ischemic heart failure. However, SMC-EPC bi-level cell sheet transplantation attenuated myocardial dysfunction, as evidenced by a slight increase in LV ejection fraction (untreated vs. cell sheet vs. sham, 38 ± 1% vs. 43 ± 3% vs. 66 ± 1%, respectively, *p* < 0.0001). The cell sheet transplantation also induced significant reverse LV remodeling, as supported by decreases in LV dimensions [LV end-diastolic volume (LVEDV) in untreated vs. cell sheet vs. sham, 585 ± 27 µL vs. 443 ± 30 µL vs. 275 ± 8 µL, respectively, *p* < 0.0001; LV end-systolic volume (LVESV) in untreated vs. cell sheet vs. sham, 365 ± 21 µL vs. 263 ± 26 µL vs. 93 ± 4 µL, respectively, *p* < 0.0001] and LV mass (untreated vs. cell sheet vs. sham, 385 ± 14 mg vs. 353 ± 24 mg vs. 278 ± 7 mg, respectively, *p* < 0.0001).

Similarly, as a part of regional functional assessment at the level of mid-LV, SMC-EPC bi-level cell sheet transplantation improved fractional shortening (untreated vs. cell sheet vs. sham, 20 ± 1% vs. 24 ± 2% vs. 34 ± 1%, respectively, *p* < 0.0001), and decreased in LV diameters [LV end-diastolic diameter (LVEDD) in untreated vs. cell sheet vs. sham, 8.8 ± 0.2 mm vs. 8.2 ± 0.2 mm vs. 6.4 ± 0.1 mm, respectively, *p* < 0.0001; LV end-systolic diameter (LVESD) in untreated vs. cell sheet vs. sham, 7.1 ± 0.2 mm vs. 6.3 ± 0.3 mm vs. 4.2 ± 0.1 mm, respectively, *p* < 0.0001].

Myocardial viability, as calculated by tracing MEMRI enhancement, demonstrated the recovery of the viable area following SMC-EPC bi-level cell sheet transplantation (untreated vs. cell sheet vs. sham, 73% ± 2% vs. 77% ± 3% vs. 100%, respectively, *p* < 0.0001). All cardiac MRI and MEMRI measurements are summarized in Table 1.

**Table 1:** Cardiac MRI and MEMRI measurements showing changes in LV function and myocardial viability.

**Cardiac MRI**							
	Untreated (n=23)	Cell sheet (n=15)	Sham (n=20)	ANOVA	Untreated versus Cell sheet	Cell sheet versus Sham	Untreated versus Sham
LVEDV (µL)	585 ± 27	443 ± 30	275 ± 8	<0.0001	0.0001	<0.0001	<0.0001
LVESV (µL)	365 ± 21	263 ± 26	93 ± 4	<0.0001	0.0005	<0.0001	<0.0001
LVEF (%)	38 ± 1	43 ± 3	66 ± 1	<0.0001	0.0546	<0.0001	<0.0001
LV mass (mg)	385 ± 14	353 ± 24	278 ± 7	<0.0001	0.1406	0.0014	<0.0001
FS (%)	20 ± 1	24 ± 2	34 ± 1	<0.0001	0.0136	<0.0001	<0.0001
LVEDD (mm)	8.8 ± 0.2	8.2 ± 0.2	6.4 ± 0.1	<0.0001	0.009	<0.0001	<0.0001
LVESD (mm)	7.1 ± 0.2	6.3 ± 0.3	4.2 ± 0.1	<0.0001	0.0033	<0.0001	<0.0001

**MEMRI**							
	Untreated (n=23)	Cell sheet (n=15)	Sham (n=20)	Wilcoxon	Untreated versus Cell sheet	Cell sheet versus Sham	Untreated versus Sham
Myocardial viability (%)	73 ± 2	77 ± 3	100 ± 0	<0.0001	0.4553	<0.0001	<0.0001

MRI = magnetic resonance imaging, LVEDV = left ventricular end-diastolic volume, LVESV = left ventricular end-systolic volume, LVEF = left ventricular ejection fraction, FS = fractional shortening, LVEDD = left ventricular end-diastolic diameter, LVESD = left ventricular end-systolic diameter. For comparisons among three groups, normally distributed variables were compared using the student *t-test* following Analysis of Variance (ANOVA).

MEMRI = manganese-enhanced MRI. For comparisons among three groups, non-normally distributed variables were compared with Wilcoxon Each Pairs test following the Wilcoxon / Kruskal–Wallis test.

### Longitudinal changes in cell engraftment following SMC-EPC cell sheet transplantation

The cell engraftment ratio was quantitatively calculated using ultrasmall superparamagnetic iron oxide (USPIO)-enhanced MRI to assess the longitudinal change following SMC-EPC bi-level cell sheet transplantation (Fig. 4C). Seven days after cell-sheet transplantation, the cell engraftment ratio was maintained as high as 92.4% of the baseline at day 0. After this, cell engraftment slowly declined until day 14, when the ratio decreased to 72.5% of the baseline. However, this ratio was maintained at approximately 70% of the baseline from day 14 to day 42. Eventually, this ratio remained as high as 56.3% of the baseline on day 56, indicating that more than half of the iron-labeled cells could still be detected at 8 weeks post-transplantation (Fig. 4D).

### SMC-EPC cell sheet induced biological adhesion and vascular development pathways

**Figure 5:**
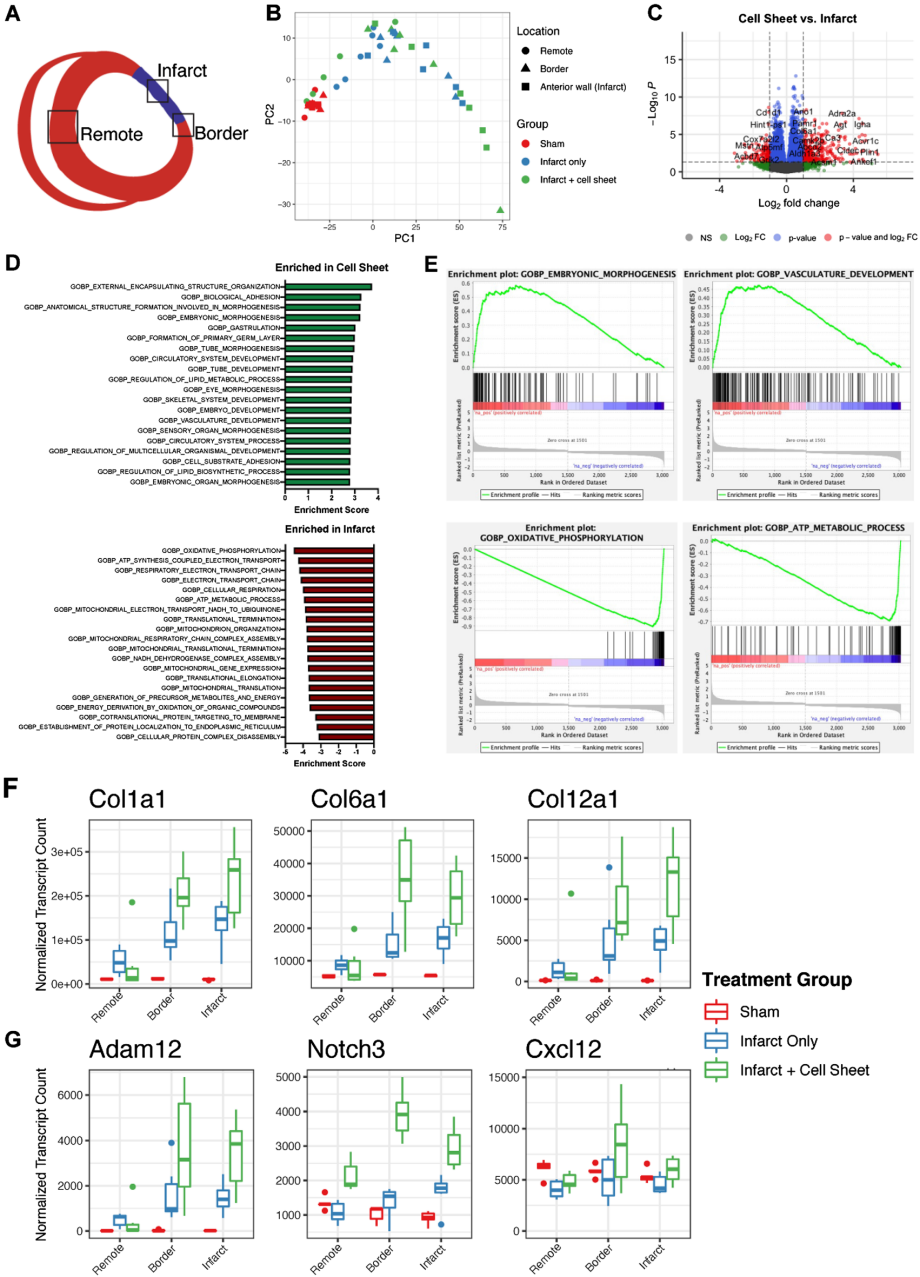
Bulk RNA sequencing to comprehensively evaluate the effect of SMC)- EPC bi-level cell sheet transplantation on native tissue biological process following myocardial infarction. **(A)** Heart tissue was collected 7 days after surgery and the left ventricle was dissected into three zones encompassing the infarct, infarct border zone, and unaffected (remote). **(B)** Principal component analysis of the individual samples highlighted variable signatures in the anterior LV (infarct) and border zone regions between the cell sheet and untreated myocardial infarction models. **(C)** We identified 114 significantly enriched processes in cell sheet-treated hearts, including multiple pathways related to primitive morphogenesis, biological adhesion, and vasculature development. **(D, E)** Infarct samples showed significant enrichment for pathways related to mitochondrial energetics, oxidative phosphorylation, and protein translation. **(F)** Within the adhesion pathway, we found increased expression of critical ECM components following cell sheet treatment, including several collagen VI isoforms and multiple collagen subtypes associated with cardiac development and response to injury (*Col12a1* and *Col1a1*). Each of these genes had low expression in sham samples and the remote, non-infarcted LV but showed enhanced expression following infarction, which was further enriched in cell sheet-treated samples, suggesting amplification of native ECM remodeling processes. **(G)** Within the vasculature development pathway, we observed heightened expression of *Ndnf* and *Adam12*, genes with known roles in angiogenic processes following cell sheet transplantation. Boxes denote interquartile range (IQR), the line represents the median, whiskers extend 1.5 IQR from box hinges.

To comprehensively evaluate the effect of SMC-EPC bi-level cell sheet transplantation on native tissue biological processes following MI, we processed heart tissue samples for bulk RNA sequencing one week after implantation. The RNA samples from cell sheet-treated MI groups were compared with those from the untreated MI groups and sham controls (n = 5–6 animals per group). To provide spatial context to the effect of cell sheet transplantation, separate samples encompassing the anterior LV (infarct zone), lateral wall (border zone), and ventricular septum (remote) were used from each animal (Fig. 5A). Principal component analysis of the individual samples showed variable signatures in the anterior LV and border zone regions between the cell sheet-transplanted group and the MI only group (the untreated MI or the infarct-only group), suggesting substantial transcriptomic differences between these groups, whereas the sham group clustered within a tight PC spectrum, indicating minimal gene expression variation due to anatomic segmentation **(**Fig. 5B). To identify changes due to cell sheet transplantation that may contribute to improvement in post-infarction myocardial function and ventricular remodeling, we directly compared the infarct and border zones of the cells between the cell sheet-treated group and the untreated MI groups. This analysis identified 3,160 differentially expressed genes (DEGs), including 1,625 enriched in cell sheet-treated hearts and 1,535 enriched in infarct-only samples. We then performed gene set enrichment analysis by ranking the DEGs in descending order of fold change and by analyzing this rank list to identify enriched biological processes in annotated gene ontology (GO)^18,19^.

We identified 114 significantly enriched processes in the cell sheet treated group, including pathways related to embryogenic morphogenesis, biological adhesion, and vasculature development, whereas the infarct-only group showed enrichment for pathways related to mitochondrial energetics, oxidative phosphorylation, and protein translation (Fig. 5C–E). Because we hypothesized that cell–ECM interaction and angiogenesis play critical roles in post-infarction cardiac performance, we further analyzed biological adhesion and vasculature development pathways for genes of interest in terms of spatial expression. For the adhesion pathway, we found increased expression of critical ECM components, including collagen VI isoforms and collagen subtypes associated with cardiac development and response to injury in the cell sheet-treated group (*Col12a1* and *Col1a1*) **(**Fig. 5F**)**^20^. The expression of each of these genes was downregulated in sham samples and the remote, non-infarcted LV but was upregulated in the untreated MI group, which were further enriched in the cell sheet treated group, thus suggestive of the enhancement of native ECM remodeling processes **(**Fig. 5F**)**. For the vasculature development pathway, *Ndnf*^21^ and *Adam12*^22^, associated with angiogenic processes, were upregulated in the cell sheet-treated group **(**Fig. 5G**)**. Other upregulated genes were *Notch3,* a mediator of angiogenesis and vascular mural cell investment^23^, and *Cxcl12*, a critical signaling molecule in the recruitment of endothelial progenitor cells^24,25^ in other preclinical models of post-MI remodeling, particularly in the border region (Fig. 5G). Collectively, these pathways establish transcriptome-wide changes in the left ventricular response to acute ischemia, indicating that the bi-level cell sheets gradually promote productive remodeling and prevent pathological ventricular dilation via these pathways and genes.

### SMC-EPC bi-level cell sheet induced morphologic reverse remodeling

**Figure 6:**
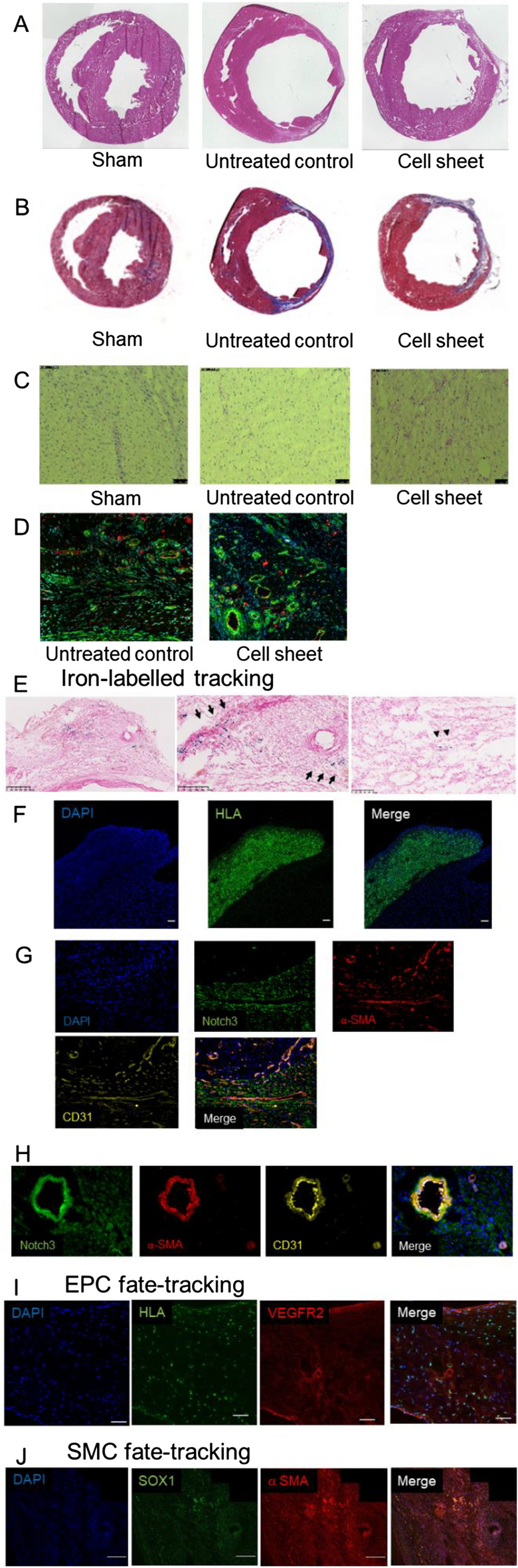
Histological Assessment. **(A)** Representative images of hematoxylin–eosin stained myocardial sections along the short axis for untreated control, smooth muscle cell (SMC)-endothelial progenitor cell (EPC) bi-level cell sheet, and sham surgery control groups. **(B)** Representative images of Masson’s Trichrome stained myocardial sections of each animal group. **(C)** The representative periodic acid-Schiff stained myocardial section along the short axis in the remote non-infarct myocardium for untreated control, SMC-EPC bi-level cell sheet, and sham surgery control groups. Scale bar = 50 μm. **(D)** Representative vWF and alpha-smooth muscle actin (α-SMA) staining of the border zone myocardium for untreated control, SMC-EPC bi-level cell sheet, and sham surgery control groups. Red indicates von Willebrand factor (vWF); green, α-SMA; blue, nuclei. **(E)** Representative images of Prussian blue-stained myocardial sections along the short axis 8 weeks after iron-labeled SMC-EPC bi-level cell-sheet transplantation. Iron-positive cells were detected in the attached cell sheet (black arrow) and host myocardium (arrowhead). **(F)** Immediately after cell sheet transplantation, immunoconfocal microscopy demonstrated human leukocyte antigen (HLA)-positive cell sheet constructs attached to the epicardial membrane of the host myocardium. Green indicates HLA; blue, nuclei. Scale bar = 50 μm. **(G)** One week after cell sheet transplantation, immunoconfocal microscopy showed CD31, α-SMA-positive, and Notch3-positive cells in the host myocardium, whereas CD31 and α-SMA-positive, but Notch3-negative cell sheet construct was attached to the host myocardium. Green indicates Notch3; red, α-SMA; yellow, CD31; blue, nuclei. **(H)** Representative Notch3, CD31, and α-SMA staining of the border zone myocardium for the SMC-EPC bi-level cell sheet transplanted group. Green indicates Notch3; red, α-SMA; yellow, CD31; blue, nuclei. **(I)** Fate tracking of transplanted human-derived EPCs performed with anti-HLA and anti-vascular endothelial growth factor receptor 2 (VEGFR2). Immunostaining for HLA and VEGFR2 showed that transplanted EPCs over the border zone contributed to myocardium neovascularization 8 weeks after transplantation. Green indicates HLA; red, VEGFR2; blue, nuclei. Scale bar = 50 μm. **(J)** Fate tracking of transplanted human male-derived SMCs performed with fluorescence in-situ hybridization to identify male SMCs in the female recipient for SRY box transcription factor 1 (SOX1) gene of male cells. Immunostaining with an antibody against SOX1 and α-SMA demonstrated that SOX1-positive SMCs originating from the transplanted bi-level cell sheet migrated into the treated myocardial tissues and contributed partly to myocardium neovascularization 8 weeks after transplantation. Green indicates SOX1; red, α-SMA; blue, nuclei. Scale bar = 200 μm.

A subset of hearts was explanted for analysis 8 weeks after MI surgery and cell sheet transplantation and assessed at a single anatomical level as a control comparison. The sham hearts preserved tissue architecture when examined microscopically using H&E staining (Fig. 6A). However, untreated MI hearts revealed widespread areas of acute inflammatory infiltration with evidence of severe ischemic necrosis and tissue edema within the myocardium. Severe LV wall thinning and dilation was also notable in the untreated MI group. However, the LV myocardial structure was superiorly maintained in the cell sheet-treated MI hearts with decreased LV volume, slight wall thickening, and reduced scar size, further confirmed by Masson’s Trichrome staining (Infarct Size; cell sheet vs. untreated, 14 ± 1% vs. 23 ± 1%, respectively, *p* = 0.03) (Fig. 6B). In addition, the SMC-EPC bi-level cell sheet attenuated cellular hypertrophy, as indicated by cellular diameter (cell sheet vs. untreated vs. sham, 20 ± 0.7 μm vs. 23 ± 0.7 μm vs. 16.5 ± 0.8 μm, respectively, *p* = *0.0003*) (Fig. 6C).

### SMC-EPC bi-level cell sheet enhanced intramyocardial arterial density

Eight weeks after MI, greater vessel density (vWF** + **/α-SMA** + **vessels) was observed in the border zone myocardium in the cell sheet-treated animals compared to that in the untreated MIs (cell sheet vs. untreated, 4.8 ± 1.4/mm^2^ vs. 3.2 ± 1.1/mm^2^, respectively) (Fig. 6D), suggesting enhanced angiogenesis in the cell sheet group.

### Engraftment of transplanted iron-labeled EPCs and SMCs in the host myocardium

Eight weeks post-cell sheet-treated hearts were stained with Prussian blue staining to confirm the engraftment of transplanted iron-labeled EPCs and SMCs. As expected, the iron-positive signals were detected in the cell sheet remaining on the epicardium of the hearts. Furthermore, these signals were detected in the host epicardial-myocardial border and further down in the myocardium (Fig. 6E), which supports the improved retention and engraftment of transplanted cells observed in USPIO-enhanced MRIs and suggests the migration of transplanted cells to the host myocardium, particularly to the border zone.

### Engraftment of bi-level SMC-EPC cell sheets and their contribution to angiogenesis in the host myocardium

The engraftment and contribution of the transplanted human-derived EPCs and SMCs to neovascularization in the host myocardium were further assessed using confocal immunofluorescence microscopy. To make cell fate tracking more straightforward, we constructed the cell sheet in a sex-specific manner using SMCs from a human male donor and EPCs from a human female donor and transplanted this cell sheet onto a female rat. We first confirmed that the rat heart immediately explanted following cell sheet transplantation showed positivity for HLA antibody only in the human-derived cell sheet attached to the host epicardium but not in the heart (Fig. 6F). Then, we further tracked the fate of EPCs and SMCs from the cell sheet using species- and sex-specific antibodies, HLA and VEGFR2 for EPCs and SOX1 and a-SMA for SMCs. Eight weeks after cell sheet transplantation, the co-localization of HLA and VEGFR2 was seen in some vessel-like structures in the border zone of the host myocardium (Fig. 6I). Similarly, a-SMA + /SOX + cells were detected around the vessels in the border zone area (Fig. 6J). These findings indicate that transplanted EPCs and SMCs may contribute to forming new blood vessels in the host myocardium.

### The contribution of bi-level SMC-EPC cell sheets to forming functional blood vessels may be triggered by the activation of the Notch signaling pathway-

The immunohistochemical assessment was performed to verify the upregulated Notch3 expression in the one-week post-cell sheet treated MI hearts from RNA sequencing. One week after cell sheet transplantation, increased CD31 + /a-SMA + /Notch3 + signals were detected in the host epi-myocardium **(**Fig. 6G**).** Interestingly, the a-SMA + /CD31 + vessel-like structures were not only formed in the cell sheet, but their number appeared to increase while moving toward the epi-myocardium border and myocardium. However, the Notch3 signal only appeared in the host heart but not in the cell sheet. Importantly, structurally mature CD31 + /a-SMA + /Notch3 + blood vessels were observed in the border zone myocardium **(**Fig. 6H).

## Discussion

Developing novel therapies that are reproducible and clinically translatable is critical if one hopes to successfully transition research from the bench to the bedside. Although our previous study demonstrated that a SMC-EPC cell sheet is a potent angiogenic construct, the use of rat-derived cells precluded clinical translation. The use of human-derived cell sheets in an athymic rat model of ischemic cardiomyopathy in our current study thus brings us one step closer to clinical translation. Moreover, a confluent bi-level cell sheet engineered in an enzyme-free manner retained ECM and essential interactions between cells, allowing for 3D culture development, advantageous to other scaffold-based cell approaches that fail to mimic the natural microenvironment of tissues and cause undesirable toxicity and immune rejection toward xenogenic materials.

The superior therapeutic effects of cell sheet transplantation to other cell delivery approaches, such as direct cell injection into the myocardium or blood circulation, are more evident by the improved cell retention and engraftment, producing more targeted cardioprotective effects on the heart. Our longitudinal tracking of iron-labeled cell sheets using USPIO-enhanced MRI showed that more than half of the iron signal could still be detected from the hearts eight weeks after transplantation. In addition, we observed the increased density of structurally mature vessel-like structures and myocardial viability in the ischemic border zone myocardium following cell sheet transplantation. Our histological assessment showed that the signals from the human-specific antibodies, HLA and SOX1, which were used to detect EPCs and SMCs, colocalized with some of these vessel structures, suggesting the engraftment and direct contribution of transplanted cells to angiogenesis in the host myocardium. Furthermore, cardiac MRI showed enhanced myocardial function recovery and limited adverse ventricular remodeling with improved cardiac function in the cell sheet-treated MI groups compared to the untreated MI groups. These findings suggest that the SMC-EPC bi-level cell sheet may improve adverse cardiac remodeling and function after ischemic injury by enhancing angiogenesis in the host myocardium.

Our in-vitro study provides compelling evidence for the angiogenesis-driven cardioprotective effect of the SMC-EPC bi-level cell sheet and its potential therapeutic mechanism. When EPCs were cultured in the SMC-conditioned culture medium, they showed a significantly enhanced angiogenic capacity than when cultured in a fresh EPC culture medium, making the paracrine mechanism a likely mediator of the effects found in this study. Angiogenesis generally refers to the formation of new blood vessels from pre-existing vasculature^26,27^. The mechanisms of angiogenesis induced by cellular therapies remain mysterious for the most part yet are surmised to involve the production of cytokines and growth factors and thus require the coordination of a series of cellular processes^28^. CXCL12, also known as stromal cell-derived factor 1 (SDF-1), binds selectively to two chemokine receptors, CXCR4 and ACKR3^29,30^. Many studies emphasize the importance of CXCR4/CXCL12 chemokine signaling in the maturation of blood vessels. For example, a study showed that CXCL12 and CXCR4 mutant embryos displayed a poorly formed coronary arterial network that compromised heart growth and function, leading to embryo lethality^31^. Another study suggested that CXCR4/CXCL12 signaling in the arterial endothelium promotes PDGFB ligand expression, driving arterial vascular SMC recruitment. These results align with the previous findings of our group that CXCL12 induced neovasculogenesis in the ischemic border zone, improved ventricular remodeling and function after MI ^32,33^, and facilitated the formation of the collateral artery network and functional recovery of the heart ^34^. Furthermore, the CXCR4/CXCL12 axis is known to play a central role in the mobilization of stem cells and their homing to ischemic tissues ^25,35^, which may explain the migration of transplanted cells in the ischemic myocardium of the recipient observed in this study. These ideas support our finding of the upregulated expression of CXCL12 and the formation of structurally-organized blood vessels in the ischemic border zone myocardium, and the more significant upregulation of this gene following SMC-EPC bi-level cell sheet transplantation suggests the contribution of transplanted cells in angiogenesis activated by this signaling pathway.

Along with CXCL12, the expression of Notch3 was also significantly upregulated in the ischemic border zone myocardium, which is supported by the previous reports that Notch signaling is implicated in arteriogenesis^36^ and intersects with hypoxic signalings, such as HIF1α^37^. Studies suggest that Notch signaling plays a vital role in angiogenesis through interactions with Notch ligands and crosstalk with other pathways like CXCR4/CXCL12. The cooperation between Notch and CXCR4/CXCL12 has been reported in tumor angiogenesis and hematological malignancies^38,39^. Furthermore, Notch is known to be the main factor in the maturation and hierarchical organization of arterial vessels. Notch3 not only functions to regulate arterial differentiation and maturation^40^ but also controls the involvement and stability of mural cells and pericytes contributing to functional blood vessels^40,41^. In addition, studies support the role of Notch3 in mediating mechanotransduction^24,41,42^. When in a contractile condition, vascular SMCs regulate vascular tone in resistance arteries, but upon mechanical stress, they show phenotypic plasticity by switching to de-differentiated phenotypes, contributing to vascular growth and remodeling^42^.

The Notch3 expression was upregulated to a greater extent in response to cell sheet transplantation after ischemic injury, especially in the ischemic border zone myocardium, which was further verified microscopically by an immunohistochemical assessment. One week after cell sheet treatment, a significant number of a-SMA + /CD31 + vessel-like structures were formed in the cell sheet that remained on the epicardium, and their number appeared to increase as moving toward the host myocardium in the ischemic border zone region. Interestingly, the Notch3 signal co-localized with the a-SMA + /CD31 + vessel-like structure in the host myocardium but not in the attached cell sheet. This finding may suggest that the activation of the CXCR4/CXCL12 signaling pathway in the host myocardium is necessary for Notch3 to initiate its contribution to angiogenesis.

Based on the previous studies and our findings, we could propose a plausible mechanism of angiogenesis-induced cardioprotective effects of the SMC-EPC bi-level cell sheet after MI: the transplanted EPCs and SMCs were recruited to the ischemic host myocardium due to the activation of the CXCR4/CXCL12 and Notch signaling pathways as an adaptive response to hypoxia, and the essential angiogenic factors released from EPCs, supported by the increased expression of Notch3 in engrafted SMCs or pre-existing vascular SMCs in the host myocardium, contributed to forming structurally mature new blood vessels, thereby improving ventricular remodeling and function.

Finally, our data showed the significant upregulation of genes related to biological adhesion and ECM pathways, primarily type VI collagen isomers and collagen subtypes. Type VI collagen not only aids cell attachment and connects with the surrounding matrix but is also known to act as an early sensor of the injury/repair response^43,44^. One study reported that collagen VI forms an anchoring meshwork that connects the interstitial matrix to the vascular basement membrane and contributes to angiogenesis by bringing cells to the surrounding environment and organizing the three-dimensional tissue architecture of the vasculature through direct interaction with collagen VI^45–47^. These biological characteristics of type VI collagen could have similarly contributed to the improved engraftment of transplanted cells and their involvement in neovascularization in the myocardium of the recipient, like CXCR4/CXCL12 and Notch signaling pathways.

Elucidating the whole mechanism by which the SMC-EPC bi-level cell sheets limit adverse cardiac remodeling is difficult. The present study identified the reverse ventricular remodeling process, such as LV volume and LV mass, as measured using cardiac MRI images following cell sheet transplantation. In addition, cell sheet transplantation attenuated cellular hypertrophy in the non-infarcted remote myocardium, as observed microscopically. We attempted to explain the correlation between these two results of ventricular reverse remodeling and myocardial reverse remodeling. First, neovascularization and the following improved myocardial viability on the targeted myocardial territory could reduce the number of necrotic/apoptotic cardiomyocytes. Next, the reduced accumulation of fibrous components would inhibit the thinning of the left ventricular wall^48,49^. Furthermore, the preserved wall thickness could reduce wall stress of the left ventricle, theoretically leading to reduced LV volume, known as adverse cardiac remodeling^50^. On the other hand, our data, together with the theory of Grossman et al. on volume-related cellular hypertrophy (i.e., cell thickening and elongation)^51^, suggest that abolishing volume overload could activate cellular and extracellular mechanisms that modify myocardial structural remodeling. Moreover, our bulk RNA sequencing indicated that the native ECM remodeling processes were enhanced after cell sheet transplantation. Thus, we speculated that the release from volume overload could initiate a subsequent adjustable response in the molecular signals that result in the regression of myocardial hypertrophy and extracellular matrix turnover^52^.

Cell engraftment is another critical aspect of myocardial regeneration. The present study focused on serial changes in a detailed fashion using a USPIO-enhanced MRI study on cell engraftment following cell-sheet transplantation. With regard to cell engraftment, the data demonstrated prolonged cell retention of the transplanted SMC-EPC cell sheets on the myocardium. The potential advantages of cell-sheet technology have been reported to include the delivery of a larger number of transplanted cells and the integration with native tissues without destroying the cell–cell or cell–ECM adhesions in the cell sheet^53,54^. In addition, the degree of neovascularization in the transplanted myocardium and subsequent myocardial inflammation after cell sheet transplantation affect the retention and engraftment of transplanted cells in cell sheet therapy^55,56^. Based on these findings and the significant findings of long-standing cell retention and the recruitment of transplanted cells from the SMC-EPC bi-level cell sheet, the SMC-EPC cell sheet might have improved the hypoxic environment in the transplanted area to a greater degree, thus potentially improving the initial and long-term cell engraftment through a higher expression of associated genes^57^.

Our study, despite intuitive findings, has several limitations that can be improved with further investigation. Our USPIO-enhanced MRI showed that more than half of the iron signal from the iron-labeled cell sheet was detected in vivo eight weeks after cell sheet transplantation compared to the baseline. However, we cannot exclude the possibility of having a false-positive signal from the non-viable cells. The long-lasting hypo-intensities in cardiac magnetic resonance (CMR) are known to originate from superparamagnetic iron oxide (SPIO)-engulfed macrophages during long-term stem cell tracking^58,59^. As discussed in several studies, extracellular iron particles can also cause a CMR hypo-intensive signal in the long-term monitoring of transplanted stem cells after injury, possibly giving a false-positive signal^58^. Considering the ischemic injury severity and duration of cell sheet transplantation in our animals, these explanations are plausible and could confound our USPIO-cell tracking results. Thus, a future study demonstrating macrophage infiltration or false-positive signal from free USPIO particles is recommended to address this issue. For example, co-localization of immunohistochemistry and Prussian blue could help identify whether the signal observed is from the transplanted cells, macrophages that have phagocytosed the iron particles^59^, or free USPIOs present in the interstitial space after cell death.

Our narrow focus on angiogenesis also limits our study. Post-infarction left ventricular remodeling is a complex process involving changes in structure, shape, and topography at the global level and changes in cardiac cells at the cellular and subcellular levels that negatively impact function^60,61^. The pathological response to the ischemic injury can be described in three main phases (inflammatory, proliferative, and adverse remodeling^62^. The lack of blood supply and oxygen and nutrient uptake due to ischemia first causes millions of cardiomyocytes (CMs) to die within a few hours after ischemic injury. Next, the apoptosis occurs for days, followed by a surge of inflammatory response in the ischemic site for weeks. Then, the activated myofibroblasts start depositing extracellular matrix collagen, resulting in the formation of an irreversible scar. Finally, the myocardial necrosis causes a disproportionate ventricular wall thinning of the infarct region of the heart, which cannot withstand the pressure and volume load on the heart in the same manner as the other part of the healthy myocardium, leading to dilation of the chamber^62,63^.

PAS staining showed that the SMC-EPC bi-level cell sheet attenuated cellular hypertrophy, as indicated by a decrease in cellular diameter (cell sheet vs. untreated vs. sham, 20 ± 0.7 μm vs. 23 ± 0.7 μm vs. 16.5 ± 0.8 μm, respectively), yet our cell sheet treatment may have a lesser effect on hypertrophic remodeling than angiogenesis. Furthermore, the increase in wall thickening observed in some cell sheet-treated hearts may also be due to a girdling effect secondary to the covered cell sheet that has reduced wall stress of the LV, leading to the maintenance of the LV thickness and improvement of LV function^57,64^. As these pathophysiological processes play unique roles in post-infarction cardiac remodeling, a systematic investigation is needed to recapitulate the multifaceted and complex nature of ischemic heart disease in a specific animal model of choice^65^.

For example, the use of human stem cells in our study necessitated using the athymic animal model to prevent the immuno-rejection of xenografted cells. However, being athymic, these animals do not produce mature T cells and exhibit a reduced number of circulating lymphocytes, which may cause a differential response to the scar formation after an ischemic injury than wild-type^66^. The importance of regulatory T-cells (T-regs) in cardiac remodeling after MI was previously reported. One study showed that athymic nude mice resulted in depressed cardiac function, increased infarct size, and significant pathological events such as aneurysms and cardiac ruptures after MI. However, immune modulation by the direct injection of T-regs or overexpression of major factors secreted by T-regs reduced infarct size, preserved cardiac contractility, and increased the number of proliferating cardiomyocytes (CM)^67^. Interestingly, the authors suggested that T-regs’ effect in improving MI outcome depends on their capacity to induce CM proliferation rather than modulating the immune response. How the introduction of xenograft materials activates the innate immune response to ischemic injury in athymic animals and how this change affects wound healing progress still needs to be explored.

Our study could be further improved by providing more in-depth mechanistic insight. Although we pointed toward significant engraftment at 8 weeks post-cell sheet transplantation in our MRI data, the histological images seemed to show a lesser contribution. This observation makes the paracrine mechanism a likely mediator of the effect found here. Analysis of the secretome (including extracellular vesicles or exosomes) could have shed some light on these potential mechanisms. One significant advantage of the scaffold-free cell sheet technology is that ECM is retained, and thus the cell sheet remains intact, allowing for 3D culture development. This makes it worthwhile to study the gap junctions between adjacent cells and cell types to demonstrate how EPCs communicate and interact with SMCs to display their pro-angiogenic effect. The multi-functional integrating system of microenvironmental signals of Notch, CXCL12, and type VI collagen, such as shear stress, the composition of ECM, and angiogenesis^67^, makes it interesting to study the interaction of the EPCs and SMCs with host cardiomyocytes or other cell types such as cardiac fibroblasts or myofibroblasts of the viable myocardium. Also, alterations to both recipient tissue and transplanted cells could have been addressed by using advanced genomics technology, such as single-cell RNAseq, given the species mismatch. Again, more conventional yet informative assays could include exploring reparative and inflammatory macrophage phenotype modulation and decrease in fibrosis. Finally, although a significant step toward translation has been made in our study by creating the bi-level cell sheet with human cells and transplanting it into an athymic rat model of MI, the information is limited. Testing this system in a large animal model of MI (pigs) by immunosuppression or using pig-derived cells should improve study translatability.

In conclusion, the human-derived SMC-EPC bi-level cell sheet is a promising cellular therapy obtained from a clinically relevant source. The joint delivery of EPCs and SMCs as a scaffold-free, bi-level cell sheet platform improves cell–cell interactions and cell engraftment, allowing for enhanced localized therapeutic effects. Furthermore, the engrafted cells contribute to forming structurally mature vasculature in a host myocardium via activation of pro-angiogenic and ECM remodeling pathways, limiting adverse ventricular and myocardial remodeling after ischemic injury and thus improving ventricular function. Based on our findings from the present study, future efforts will aim to study the crosstalk between EPCs and SMCs and their interactions with host cell types and investigate the post-infarction LV remodeling more globally by looking at multiple related pathological events, which will provide more in-depth mechanistic insights.

## Methods

All methods were carried out in accordance with relevant guidelines and regulations. Our study was approved by the Institutional Review Board of Stanford University (IRB 32769), and informed consent was obtained from all subjects. The study is reported under ARRIVE guidelines.

### EPC isolation and cell culture

Blood outgrowth EPCs were isolated from healthy human donors’ peripheral blood, as previously reported^68^. Approximately 27 mL of blood was collected sterilyand diluted with phosphate-buffered saline (PBS, > 1:1). Density-gradient centrifugation was conducted by layering the blood sample over a Ficoll-Paque solution and centrifuging for 20 min at 1000 × *g* at RT. Next, the buffy coat layer containing the mononuclear fraction of peripheral blood, including EPCs, was carefully harvested and washed with Hank’s balanced salt solution (HBSS) by centrifuging for 10 min at 300 × *g at RT*. After centrifugation, the supernatant was discarded, and the pellet was resuspended in HBSS. After two washes, the isolated EPCs were resuspended in EGM-2 BulletKit (Cat. CC-3162; Lonza Inc., NJ, USA) medium and seeded on a 24-well plate previously coated with 0.2% gelatin for approximately two hours at a density of 1.5 × 10^5^ cells per well. The cells were incubated at 37 °C in a 5% CO_2_ atmosphere saturated with H_2_O, and the culture medium was changed every two days. The cobblestone morphology of the EPCs started appearing in colonies around day 14–21, which reached confluence around day 21–28, depending on donors (Fig. 1A,B).

### Trans-differentiation of SMC from MSC

Human bone marrow-derived MSCs were purchased from Lonza Inc., NJ, USA, and cultured at a density of 4–6 × 10^3^ cm^2^ in minimum essential medium alpha (MEM α; Gibco, Thermo Fisher Scientific, MA, USA) supplemented with 20% fetal bovine serum (FBS; Hyclone, GE Healthcare Life Sciences, UT, USA), 5% L-glutamine (Gibco, Life Technologies, CA, USA), and 1% penicillin/streptomycin (P/S; Gibco, Life Technologies, CA, USA) at 37 °C and 5% CO_2_. Cells were cultured for 7–10 days, and the medium was exchanged every 3–4 days. When they reached 80–85% confluency, the MSCs were lifted and cultured on a dish coated with fibronectin (BD Biosciences, CA, USA) at a density of 4–6 × 10^3^ cm^2^ in Medium 231 supplemented with smooth muscle growth supplement (SMGS; Gibco, Life Technologies, CA, USA), 10% FBS, and 1% P/S at 37 °C and 5% CO_2_ to induce SMC differentiation. SMC growth medium (Medium 231 with SMGS) was used as the nutrient medium and exchanged every 2 days. MSCs grown under SMC growth conditions were passaged several times for expansion and induction toward SMC lineage differentiation before biological tests (Fig. 1A)^18^. In addition, commercially available human aortic smooth muscle cells (AoSMC; Lonza Inc., NJ, USA) were cultured in the same medium condition at a density of 3.5 × 10^3^ cm^2^ for comparative purposes.

### Immunohistochemical assessment of the morphological characteristics of EPCs and SMCs

Approximately 40,000–60,000 cells were cultured on four-well chamber slides (Nunc™ Lab-Tek™ II Chamber Slide™ System, Cat. 154526PK; Thermo Fisher Scientific, CA, USA) and incubated overnight at 37 °C. After 24 h, the cells were washed with PBS gently in the slides and fixed with 4% PFA for 10 min at RT. Following fixation, the cells were washed with PBS and permeabilized in 0.5% PBS-Tween for 15 min at RT. After washing with PBS, the cells were blocked with 10% goat and donkey sera for an hour at RT. Following three washes, the cells were incubated with the appropriate primary antibodies overnight at 4 °C; EPCs were stained with anti-CD31 (1:200, Abcam, Cambridge, UK; Cat: ab24590), anti-CD34 (1:100, Invitrogen, CA, USA; Cat: PA5-32322), and anti-HLA (1:200, Abcam, Cambridge, UK; Cat: ab52922), and SMCs were stained with anti-SM22-α (1:100, Abcam, Cambridge, UK; Cat: ab14106), and anti-Caldesmon (1:100, Abcam, Cambridge, UK; Cat: ab212964). The cells were washed twice and stained with the appropriate secondary antibodies in the dark for an hour at 37 °C and washed again: goat anti-mouse Alexa Fluor 488 secondary antibody (1:200, Abcam, Cambridge, UK; Cat: ab150113), goat anti-rabbit Alexa Fluor 488 secondary antibody (1:200, Abcam, Cambridge, UK; Cat: ab150077), donkey anti-goat Alexa Fluor 488 secondary antibody (1:200, Abcam, Cambridge, UK; Cat: ab150129), goat anti-rat Alexa Fluor 488 secondary antibody (1:200, Abcam, Cambridge, UK; Cat: ab150165), goat anti-mouse Alexa Fluor 594 secondary antibody (1:200, Abcam, Cambridge, UK; Cat: ab150116), goat anti-rabbit Alexa Fluor 594 secondary antibody (1:200, Abcam, Cambridge, UK; Cat: ab150080), donkey anti-goat Alexa Fluor 594 secondary antibody (1:200, Abcam, Cambridge, UK; Cat: ab150132), goat anti-mouse Alexa Fluor 647 secondary antibody (1:200, Abcam, Cambridge, UK; Cat: 150115), donkey anti-rabbit Alexa Fluor 647 secondary antibody (1:200, Abcam, Cambridge, UK; Cat: 150075), and donkey anti-goat Alexa Fluor 647 secondary antibody (1:200, Abcam, Cambridge, UK; Cat: 150131). After washing, the cells were incubated with DAPI (1:100, Thermo Fisher Scientific, MA, USA; Cat: R37606) for nuclear staining in the dark for 3–5 min at RT and washed again. The slides were covered with microscope coverslips in VECTASHIELD® Antifade Mounting Medium (Vector Laboratories, H-1000) and stored in the dark at 4 °C until ready for imaging.

### Flow cytometry to evaluate EPC and SMC phenotypes

Flow cytometry was performed to identify the phenotypes of cultured EPCs and differentiated SMC using EPC and SMC markers, respectively. About 10^6^ cells were collected, washed twice with PBS, and incubated with the Zombie Aqua Fixable Viability Kit (1:20, BioLegend, CA, USA; Cat. 423101) in the dark for 30 min at RT to exclude the dead cell population. After incubation, the cells were washed twice with flow cytometry staining buffer and centrifuged for 5 min at 300 × *g*. The EPCs were then incubated with the following three surface antibodies on ice for 30 min in the dark: APC-conjugated CD31 (1:100, eBioscience, CA, USA; Cat: 17–0319-42) and PE-Cy7-conjugated CD34 (1:100, eBioscience, CA, USA; Cat: 25–0349-42). For SMCs, the cells were first fixed with a fixation medium (Reagent A of FIX & PERM™ Cell Permeabilization Kit; Thermo Fisher Scientific, MA, USA) for 15 min at RT. After two washes with staining buffer, the SMCs were incubated with a permeabilization medium (Reagent B of FIX & PERM™ Cell Permeabilization Kit; Thermo Fisher Scientific, MA, USA) and three intracellular antibodies on ice for 30 min in the dark: PE-conjugated α-SMA (1:100, Abcam, Cambridge, UK; Cat: ab209435), APC-conjugated SM22-α (1:100, Abcam, Cambridge, UK; Cat: ab14106 (the primary antibody was conjugated using Lightning-Link APC labeling kit; Novus Biologicals LLC, CO, USA)), and AF700-conjugated caldesmon (1:100, Novus Biologicals LLC, CO, USA; Cat: NBP2-47819AF700). The labeled EPCs and SMCs were washed twice, centrifuged for 5 min at 300 × *g*, and stored in a fresh buffer until analysis. A portion of the cell suspension was used for compensation controls. The percentage of SMCs expressing each intracellular antigen was analyzed using a Becton Dickinson LSR II flow cytometer (BD Biosciences, CA, USA). EPCs expressing each surface antigen were analyzed using Novocyte Quanteon (Agilent, CA, USA). Data analysis was performed using FlowJo vX.

### Angiogenesis assay to assess the interaction between EPCs and SMCs

To assess the synergistic effect of SMC cytokines and endothelial growth factors on the angiogenic potential of EPCs, an in vitro Matrigel angiogenesis assay was performed. EPCs and SMCs were first cultured in EGM-2 medium in separate dishes for two days. Then, the 48 h spent medium on the SMC dish that contains proteins and cytokines released from SMCs was collected and used as an “SMC-conditioned” EGM-2 medium. Growth factor-reduced Matrigel (Corning, NY, USA; Cat: 354263) was thawed overnight at 4 °C. The thawed Matrigel kept on ice was used to coat each well of a 15-well µ-Slide (Ibidi, Gräfelfing, Germany; Cat: 81507) and allowed to polymerize for 30 min at 37 °C in 5% CO_2_. Before seeding cells, we ensured that the Matrigel was evenly coated throughout the wells without bubbles. Next, 15 k EPCs were seeded in each well and cultured in one of the following experimental medium conditions: fresh EGM-2 medium (n = 24 wells) or SMC-conditioned EGM-2 medium (n = 24 wells). The well plates were incubated for 8 h at 37 °C in 5% CO_2_, and the progress of tube formation was monitored every two hours (2, 4, 6, and 8 h) post-seeding. The images were then analyzed using the Angiogenesis Analyzer ImageJ plugin^69^. For analysis, values were presented as mean ± SEM, and multiple comparisons involving 24 technical replicates per condition were performed using one‐way ANOVA. A value of *P* < 0.05 was considered statistically significant.

### Creation of human-derived SMC and EPC bi-level cell sheets

Cell sheets were created on and removed from a specialized dish called Upcell (CellSeed, Tokyo, Japan). This dish is grafted covalently with a temperature-responsive polymer-poly (N-isopropyl acrylamide) that can lift adherent cells from the dish by simply undergoing an enzyme-free transformation from hydrophobic to hydrophilic upon temperature reduction (Fig. 3A), thus allowing the fabrication of three-dimensional tissue constructs from densely adherent cells without an artificial scaffold or enzymatic digestion.

A 35 mm Upcell dish was first conditioned in pre-warmed 231 medium for 30 min at 37 °C in 5% CO_2_. Then, human-origin trans-differentiated SMCs were seeded on the Upcell dish at a density of 1.5 × 10^5^/cm^2^ and cultured for 24 h at 37 °C in 5% CO_2_. After 24 h of incubation, the Upcell dish containing the confluent monolayer SMC sheet was transferred to the heating plate at 37 °C under the laminar hood. Next, human-origin EPCs suspended in a pre-warmed EGM-medium were carefully seeded at 1.5 × 10^5^/cm^2^ on top of the confluent SMC layer and cultured for an additional 24 h at 37 °C in 5% CO_2_. After 24 h of incubation, the dish containing a bilayer of two different confluent cell types was transferred at RT to lift the cells as an intact SMC-EPC bi-level cell sheet (Fig. 3B). A successful bi-level cell sheet can be prepared with the following techniques:

1. An Upcell dish, when containing cells, should always be handled at 37 °C to prevent spontaneous cell detachment due to temperature change;
2. Only healthy, proliferative, and adherent cells should be used and resuspended in a pre-warmed medium to enhance cell attachment to the Upcell dish or the first monolayer cells;
3. When seeding the cells on top of the first cell layer, cells should be dispensed drop-wise to minimize disturbing the first cell layer. Slowly dispense the cell suspension while lightly touching the pipette tip against the side of the Upcell dish instead of dropping it directly on top of the first cell layer, which could lift the cells from the first cell layer. After dispensing the suspension, swirl the plate very slowly to distribute the dispensed cells evenly.

### Using scanning electron microscopy to visualize topological variations of SMC-EPC bi-level cell sheets

We performed scanning electron microscopy (SEM) to closely observe the morphology of SMCs and EPCs and their interactions in the cell sheet (Fig. 3D–E). The bi-level cell sheet lifted from the UpCell dish was fixed with 4% paraformaldehyde and 2% glutaraldehyde in 0.1 mol/L sodium cacodylate buffer (pH 7.2) for 24 h at 4 °C, rinsed in the same buffer, and post-fixed with 1% aqueous osmium tetroxide for an hour. After dehydration in an ascending ethanol series (50%, 70%, 90%, and 100% [2x] for 10 min each), the sample was pressed onto carbon paint and sputter-coated with Au:Pd (60:40) before imaging. The SEM images were acquired using an FEI Magellan 400 XHR microscope with a beam voltage of 8 kV and a dwell time of 30 μs.

### Animal care and biosafety

Female RNU nude rats (F344/NJcl-rnu/rnu, 8 week-old, 170–200 g) were obtained from Charles River (Wilmington, MA, USA). All animals were socially housed (or in pairs) in a temperature-controlled room at approximately 22 °C with a 12 h light/dark cycle. Food and water were provided ad libitum. All animal experiments were performed following the Guide for the Care and Use of Laboratory Animals (United States National Institutes of Health, 8th Edition, 2011). In addition, all animal procedures were approved by the Institutional Animal Care and Use Committee at Stanford University (Protocols 28921 and 34273).

### Athymic rat myocardial infarction model and cell sheet transplantation

**Figure 7:**
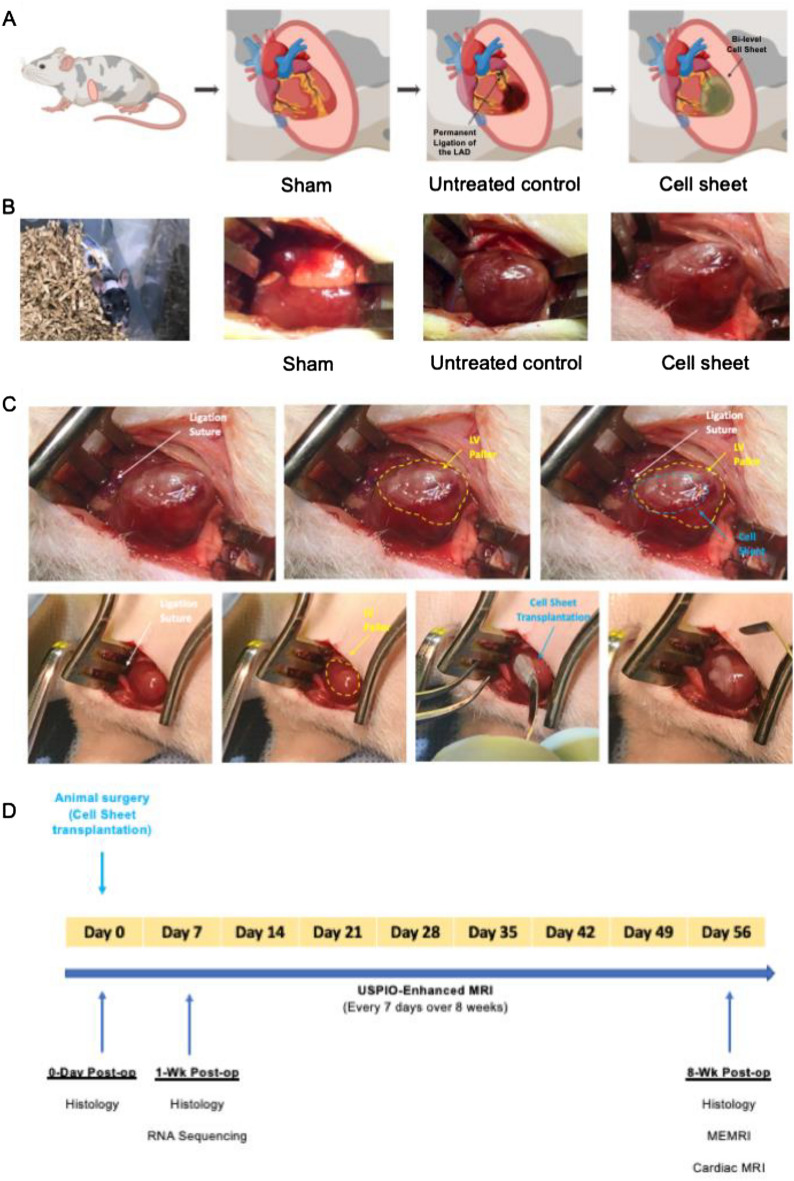
Experimental technique and protocol timeline. **(A)** A schema illustrating induction of myocardial infarction (MI) by ligating the left anterior descending coronary artery permanently and smooth muscle cell (SMC)-endothelial progenitor cell (EPC) bi-level cell sheet transplantation technique via a left thoracotomy approach. Created with BioRender.com. **(B)** Pictures of the operated rats of three groups: transplantation of SMC-EPC bi-level cell sheet (cell sheet group), no treatment (untreated control group), or a sham operation (a positive control). **(C)** Pictures of the cell sheet transplantation onto the ischemic area of the heart. **(D)** Timeline of the experimental protocol. USPIO, ultrasmall superparamagnetic iron oxide; MRI, magnetic resonance imaging; MEMRI, manganese-enhanced magnetic resonance imaging. Created with BioRender.com.

Animals were anesthetized using 2–3% inhaled isoflurane (Fluriso, VetOne, Boise, ID, USA) delivered at 1 L/min, and then endotracheally intubated with a 16 G angiocatheter. Anesthesia during mechanical ventilation was maintained using continuous 1–2% isoflurane (Harvard Apparatus VentElite, Holliston, MA, USA). Before making the incision, animals were given Bupivacaine (1 mg/kg, subcutaneously) for local pain control at the incision site. A left anterior thoracotomy was performed via the fourth intercostal space, followed by an opening of the pericardium to expose the heart. Once the left anterior descending coronary artery (LAD) was visualized, a proximal region of the LAD (or 1 mm below the left atrial appendage) was permanently occluded using a 6–0 polypropylene suture (Fig. 7A). Consistently sized infarcts were confirmed by visually assessing the area of the resulting LV pallor. We utilized an established ischemic cardiomyopathy model with a resultant MI encompassing 40% of the left ventricle. Each MI-operated rat was randomly allocated to one of the following two groups by using the standard = RAND() function in Microsoft Excel: (1) transplantation of SMC-EPC bi-level cell sheet (cell sheet group, n = 32, 2 animals died during operation) or (2) no treatment (untreated control group, n = 52, 18 animals died during or post-operation). These two groups were compared to (3) Sham controls (positive control, n = 32, 1 animal died post-operation) (Fig. 7B). For the cell sheet group, the SMC-EPC bi-level cell sheet was directly placed on the epicardium of the ischemic area following a 15 min post-infarct period (Fig. 7C). The cell sheet transplanted heart was monitored for another 15 min to ensure its attachment to the beating heart before closing the chest. The chest was closed in layers using interrupted 5–0 polypropylene sutures. After completion of the surgery, the animals were extubated and recovered. Postoperative analgesia was achieved using Buprenorphine SR (1.2 mg/kg, subcutaneously). Animal exclusion criteria during the perioperative setting include the absence of or minimal pallor of the myocardium following LAD ligation, ventilator dependency or labored spontaneous breathing, uncontrolled bleeding, and irreversible damage to the heart, lung, or other organs. All experiments involved in this study were performed blindly. The surgeon was blinded with the animals’ weights, cage information and the treatments the animals received throughout the surgical procedures (both survival and terminal). All other investigators were also blinded to this information and used the coded IDs for their experimental analyses. The IDs were decoded only after all data were collected and analyzed.

### Cardiac MRI for evaluating ventricular remodeling and cardiac function, mass, and myocardial viability

Cardiac MRI was performed at 8 weeks post-operation using a Signa 3 T EXCITE scanner (GE Healthcare, WI, USA) and a phased array 4 channel surface coil (Rapid MR international)^16,70^. The animals were anesthetized using 1.0–3.0% isoflurane throughout the scan. ECG gating and respiratory and body temperatures were monitored using PC-SAM (SA Instruments Inc., NY, USA). The LV function was evaluated on short-axis serial slices obtained by an ECG-triggered fast spoiled gradient-recalled (FSPGR) sequence. Nonviable and viable myocardium were discriminated using MEMRI, which was performed using an ECG-triggered IR-FSPGR sequence approximately 60 min after the subjects were given an intraperitoneal injection of 10 mL/kg of EVP103 (Eagle Vision Pharmaceutical)^71,72^. The LV contours were traced manually to calculate LVEDV, LVESV, and LV ejection fraction (LVEF). Left ventricular end-diastolic diameter (LVEDD) and left ventricular end-systolic diameter (LVESD) at the level of the papillary muscles (mid-LV level) were measured. Fractional shortening (FS) was calculated as a parameter of regional LV function: FS (%) = (LVEDD—LVESD)/LVEDD. A manganese-based contrast agent (EVP 103) was taken up by L-type calcium channels to confer T1-shortening and positive signals on the viable myocardium. Myocardial viability was calculated by tracing MEMRI enhancement. MEMRI viable myocardial volume (%) = (MEMRI enhancement volume × 100)/total LV mass volume.

### USPIO-enhanced MRI for assessing engraftment of transplanted donor cells

USPIO-enhanced MRI was performed to evaluate the retention and engraftment of transplanted SMC-EPC cell sheets on the host tissue. SMCs and EPCs were first magnetically labeled before cell sheet creation. Next, an adherent monolayer of differentiated SMCs or EPCs was cultured and lifted when it reached 80–90% confluence and resuspended in serum-free Dulbecco's modified Eagle’s media at a density of 4 × 10^6^ cells/mL for labeling. Each cell type suspension was mixed with protamine (60 µg mL^−1^) and ferumoxytol (50 µg mL^−1^) from stock drugs and incubated for 2–4 h at RT. After the iron labeling, an equal amount of complete medium containing 10–20% FBS was added to the cells and incubated overnight at RT. The iron-labeled cells were then washed with PBS and used to create bi-level cell sheets using the method described above.

Iron labeling was confirmed using USPIO with FDA-approved ferumoxytol injection (Feraheme; 1000 mg/mL; AMAG Pharmaceuticals, Inc., MA, USA). After cell sheet transplantation, cardiac MRI was performed using a Signa 3 T EXCITE scanner (GE Healthcare, IL, USA) and a phased array 4 channel surface coil (Rapid MR International, LLC, OH, USA) weekly over 8 weeks until euthanasia. The animals were anesthetized using 1.0–3.0% isoflurane throughout the scan. ECG gating and respiratory and body temperatures were monitored using PC-SAM (SA Instruments Inc., Maharashtra, India). The iron-labeled cells in the transplanted cell sheet were detected as dephasing signal loss on gradient-echo (GRE) sequence on days 1, 7, 14, 28, 42, and 56 post-transplantation. The GRE images were obtained on LV short-axis planes to cover the whole heart (flip angle = 35°, TR = 1 RR, TE = 9 ms, trigger delay = 12 ms, NEX = 6, matrix = 256 × 192, FOV = 4 cm, thickness = 1.5 mm, BW = 122 Hz/pixel). The USPIO-labeled areas on the hearts were measured and corrected by the USPIO density values of interest at the mid-LV level (clearly depicting the base of the papillary muscles)^73,74^.

### Heart explant and sample preparation for RNA sequencing and histology

At 1 week or 8 weeks after surgery, the animals were deeply anesthetized using 5% inhaled isoflurane. A median sternotomy was performed, and potassium chloride (1 mEq/kg) was injected into the right ventricle to induce cardiac arrest. The heart was explanted and flushed with PBS to remove blood, and excess fluid on the surface of the heart was removed using gauze. Six 1-week post-op hearts of each animal group were saved for RNA sequencing, while the remaining 1-week and 8-week post-op hearts were prepared for histology. For RNA sequencing samples, the tissue only below the ligation stitch level was used, which was further dissected into three zones encompassing the infarct, border zone, and remote of the ischemic myocardium. The tissue pieces were immediately flash-frozen in liquid nitrogen and stored at − 80 °C until use. For histology, the whole hearts were filled with optimum cutting temperature compound (OCT, Fisher HealthCare, Cat: 23730571, Houston, TX, USA), frozen in OCT using 2-methyl butane on dry ice, and stored at − 80 °C until use. A few hearts were collected at day 0 post-cell sheet treatment to serve as the baseline for histological assessment. The end-time points were illustrated in Fig. 7D as well.

### RNA sequencing to evaluate comprehensive transcriptomic characteristics

The flash-frozen tissue pieces (the infarct, border zone, and remote) were pulverized on dry ice, from which RNA was extracted using an RNeasy extraction kit (Qiagen, Germantown, MD). RNA sample integrity was ensured using a bioanalyzer. mRNA libraries were then generated using poly-A enrichment, and all samples were sequenced simultaneously using a NovaSeq 6000 device using paired-end 150-bp reads. Raw sequencing BCL files were then demultiplexed into individual FASTQ files corresponding to each sample; these files were used as input into STARAligner v.2.5.4 to simultaneously trim sequencing reads and align to the mRatBN7.2 genome assembly. The resulting aligned reads were mapped to Rattus norvegicus Annotation Release 108, and the individual sample count matrix files were generated. Next, downstream RNA sequencing analysis was performed using the DESeq2 package in R. All samples were merged into a single expression matrix data frame and normalized by sequencing depth before differential expression analysis. Finally, unsupervised differential expression testing was performed to compare cell sheet-treated to infarct only samples (or MI only samples) using the “DESeq()” command with default parameters (“glmGamPoi” fit type followed by negative binomial dispersion fitting and Wald significance test). Differentially expressed genes were then ranked by fold change and input into gene set enrichment analysis (GSEA) version 4.2.1.

### Histological assessment of infarct extent and morphological changes in host myocardium

The 8-week post-op frozen hearts were cross-sectioned along the ventricle short-axis plane with 10 μm thickness using a Leica CM3050S cryostat (Wetzlar, Germany) and stained. Hematoxylin and eosin (H&E) staining and Masson's trichrome staining were performed to demonstrate the presence of ischemic injury and evaluate the cardiac fibrosis in the remote myocardium using the reagents and instructions provided in the Thermo Scientific Shandon Rapid-Chrome H&E Frozen Section Staining Kit (Thermo Fisher Scientific, Cat: 9990001) and the Masson’s trichrome staining kit (American MasterTech, Cat: KTMTR2PT, Lodi, CA, USA), respectively. Finally, periodic acid-Schiff (PAS) staining was performed to assess hypertrophic response in the myocardium following each treatment using the PAS Stain kit (Cat No. ab150680; Abcam, Cambridge, UK). Digital photographs were taken with a NanoZoomer 2.0-RS (Hamamatsu, Japan) and Keyence BZ-X800 (Osaka, Japan) and were analyzed using the Image J software.

### Immunohistochemical assessment of mature vessel formation

The 1-week or 8- week post-op whole hearts were cryo-sectioned with 10 µm thickness and stained with anti-von Willebrand factor (vWF) (1:100, Abcam, Cambridge, UK; Cat: ab11713) and anti-α-SMA (1:100, Abcam, Cambridge, UK; Cat: ab21027) to evaluate mature vessel density. The mature vessel density was calculated as the number of positively stained vessels per heart in five randomly selected fields within the peri-infarct border zone**.** In addition, some 1-week post-op hearts were stained with anti-CD31 antibody (1:25, R&D Systems, USA; Cat: AF806), anti-a-SMA antibody (1:200, Novus Biologicals, USA; Cat: NB300-978), and anti-Notch3 antibody (1:200, Abcam, UK; Cat: ab23426) to evaluate angiogenesis in the host myocardium. Appropriate secondary antibodies were used for each primary antibody, and cell nuclei were counterstained with DAPI. Images were acquired with a fluorescence microscope (Leica, Wetzlar, Germany) and Keyence BZ-X800 (Osaka, Japan), and ImageJ was used for quantitative morphometric analyses.

### Cell fate tracking to identify transplanted donor cells in the recipient heart

We created cell sheets in a sex-specific way to track their fate on the recipient’s heart after transplantation. The cell sheets were prepared with EPCs from a human female donor and SMCs from a human male donor and transplanted onto a female athymic rat heart. The animals were sacrificed at 0-day and 8-week postoperative time points, and their hearts were sectioned as described above. The transplanted EPCs were tracked immunohistochemically with anti-HLA (1:100, Abcam, Cambridge, UK; Cat: ab52922) and anti-VEGFR2 (1:100, Abcam, Cambridge, UK; Cat: ab9530), whereas SMCs were tracked using anti-SOX1(1:100, EMD Millipore, MA, USA; Cat: 07–1673) and anti-α-SMA (1:100, Abcam, Cambridge, UK; Cat: ab21027). Cell nuclei were counterstained with DAPI.

To corroborate the fate of the transplanted bi-level cell sheets on the recipient myocardium, we labeled the cell sheets with iron, as described above, before transplantation. The iron-labeled cell sheets were transplanted onto athymic rat hearts following MI, and the hearts were collected 8 weeks postoperatively. Tissue sections were stained with the Prussian blue stain kit (Cat No. ab150674; Abcam, Cambridge, UK) according to the provided instructions to detect ferric iron in the tissues.

### Statistical analysis

Continuous variables are expressed as the mean and standard error of the mean. All the continuous variables were checked for normality using the Shapiro–Wilk test and normal probability plot. For comparisons among three groups, normally distributed variables were compared using the Student *t* test following Analysis of Variance, while nonnormally distributed variables were compared with Wilcoxon Each Pairs test following the Wilcoxon / Kruskal–Wallis test. Statistical significance was set at *p* < 0.05. All calculations were performed using JMP 9.0 (SAS Institute Inc., NC, USA).

## Data Availability

The datasets generated and/or analyzed during the current study are available in the GEO DataSets repository, https://www.ncbi.nlm.nih.gov/geo/query/acc.cgi?acc=GSE210374.
